# Topical Administration of Vitamin D2 Combined with Colloidal Silver Nanoparticles Promotes Wound Repair and Protection Against Skin Irritation and UVB Irradiation in 3D Reconstructed Human Skin Models

**DOI:** 10.3390/pharmaceutics17040472

**Published:** 2025-04-04

**Authors:** Francesca Truzzi, Camilla Tibaldi, Silvia Dilloo, Annalisa Saltari, Mitchell P. Levesque, Fabio Arcangeli, Alfredo Garzi, Giuseppe Ruggiero, Giovanni Dinelli

**Affiliations:** 1 Department of Agricultural and Food Sciences, Alma Mater Studiorum—University of Bologna, 40127 Bologna, Italy; camilla.tibaldi2@unibo.it (C.T.); silvia.dilloo2@unibo.it (S.D.); giovanni.dinelli@unibo.it (G.D.); 2 Department of Dermatology, University of Zurich Hospital, 8091 Zurich, Switzerland; annalisa.saltari@usz.ch (A.S.); mitchell.levesque@usz.ch (M.P.L.); 3 Department of Dermatology, Guglielmo Marconi University, 00193 Rome, Italy; fabio.arcangeli4@gmail.com; 4 Department of Medicine and Surgery, University of Salerno, 84084 Salerno, Italy; alfredogarzi56@gmail.com; 5 National Head of the Dermatology Study Group of the Italian Federation of General Pediatricians, 00193 Rome, Italy; ruggiero.04@libero.it

**Keywords:** vitamin D2, silver nanoparticles, skin protection, reconstructed human skin models, wound healing, skin irritation, UVB irradiation

## Abstract

**Background/Objectives**: There is a great demand for novel, multipurpose, natural skin-care products in the global skin repair and sun protection markets. Within this framework, the potential benefits of topical Vitamin D2 (VD2) administration in combination with silver nanoparticles (AgNPs) were examined. **Methods**: Evaluating the efficacy of the VD2+AgNP cream in wound healing, skin irritation and UVB irradiation protection necessitated preclinical testing using reconstructed human skin equivalent models (prepared from human foreskins) containing both a fully stratified epidermal layer and underlying dermis. **Results**: Application of the cream significantly improved wound healing by stimulating keratinocyte re-epithelialization and dermal fibroblast migration in models subjected to full-thickness (scratch and biopsy punch) wounds, compared to untreated models. The VD2+AgNP cream, administered prior to the induction of skin irritation by 5% sodium dodecyl sulfate (SDS) afforded protection by ameliorating cell viability epidermal thickness and interleukin-1alpha levels. UVB exposure (50 mJ/cm^2^) significantly reduced cell viability and epidermal thickness (associated with increased epidermal breakage), as well as basal layer Ki67 and supra-basal layer involucrin expression, compared to the CTRL sham-irradiated models. The cream administered prior to UVB irradiation (protective capacity) showed greater efficacy in minimizing epidermal damage. This was reflected by significantly higher Ki67 and involucrin expression, as well as lower epidermal breakage, compared to models where the cream was applied following UVB irradiation (curative capacity). **Conclusions**: The VD2+AgNP cream shows multipurpose potential in skin protection. The underlying molecular mechanisms remain to be investigated.

## 1. Introduction

Skin irritation (including dryness) and wound repair products currently comprise the first and second largest segments of the global skin repair market, respectively [1]. The global skin repair market is expected to increase from 80.99 billion US dollars (2023 data) to 150.30 billion US dollars by 2033 [1]. Similarly, the global sun protection market is also projected to increase from 13.11 billion US dollars to 21.05 billion US dollars over the same period [2]. These predicted increases are attributable to various factors, which include the limited efficacy of current skin repair treatments, a growing demand for natural skin products that guarantee both personal and environmental safety, and innovations in both formulation technologies and ingredients [1,2,3,4]. Of great relevance is that customers are increasingly more interested in skincare products with both medicinal and cosmetic benefits, thereby providing incentive for the development of multi-purpose products [1]. Of the widely-reported pleiotropic effects of vitamin D, the promising prospect of topical vitamin D application in skin care has gained recent attention [5].

Vitamin D, in the form of D3 (1,2 5-dihydroxyvitamin D or cholecaciferol), is produced photochemically by the action of sunlight or ultraviolet (UV) light from the precursor sterol 7-dehydrocholesterol. The latter is contained in the keratinocytes of the basal and spinous layer of the epidermis. In the epidermis, vitamin D3 (VD3) has been shown to display anti-inflammatory and anti-oxidant properties, inhibit DNA damage and stimulate DNA repair after UV exposure. In the underlying dermis, comprised mostly of fibroblasts, both vitamin D3-induced collagen production and wound healing have been demonstrated [5]. The feasibility of exogenous transdermal VD3 uptake was reported for the first time 10 years ago in a clinical pilot study to treat vitamin D deficiency [6]. However, given the hydrophobic–lipophilic properties of VD3, the limitations in VD3 drug delivery (characteristic of both supplement and topical applications) include low absorption, bioavailability and stability. As a result, the facilitated delivery of VD3 (as well as of other active ingredients) in combination with organic and inorganic nanoparticles (NPs, dimensions of 1–100 nm) has been developed and implemented by both the food and pharmaceutical industries [7]. Although advances in nanotechnology have, similarly, revolutionized the skin care industry by offering targeted delivery of active ingredients, controlled release and deep penetration [8,9], scarce attention has since been paid to the potential multipurpose benefits of administering VD3 in skin products to improve skin irritation, sun protection and wound healing.

A polymeric nanocarrier was used for the first time for the topical administration of VD3 in 2017 by Ramezanli et al. [10]. Given the continuous interest in improving the efficacy of nanocarrier systems for the drug delivery of vitamins [7], a novel nanocarrier system for VD3 using silver nanoparticles (AgNPs) was developed and tested for its effects on the viability and wound healing capacity of human keratinocytes (HaCaT cells) in vitro [11]. Combining AgNPs with VD3 for the first time, Cataldi et al. [11] showed that the wound healing of HaCaT cells was significantly improved compared to the administration of VD3 alone [12]. AgNPs, widely used in applied scientific research, were selected based on their antimicrobial and antioxidant properties [11], and are currently considered groundbreaking in wound care [9,13]. The VD3+AgNP combination was shown to accelerate the reparative activity of the HaCaT cells, involving signal transduction of the sphingolipid metabolism (SM) pathway [11]. Subsequently, using the same concentration of vitamin D2 (VD2) to substitute VD3 [11], it was shown that VD2+AgNPs induced a stronger wound-healing effect than VD3 [14]. The potential implication of VD2+AgNPs, not only in wound healing but also in irritative pathologies, was highlighted [14]. Testing the potential of VD2+AgNPs necessitates an applied approach through the use of physiologically relevant three-dimensional (3D) skin equivalents.

In accordance with the complete ban of animal testing for cosmetic purposes and the limitations imposed on animal testing for pharmaceutical purposes (wound healing) by the Principles of the Humane Experimental Technique (the 3Rs principle), it has become increasingly necessary to test formulations on artificially created skin models [15,16,17].

Given that commercially available 3D skin models are costly, the most widely employed models in use are two-dimensional (2D) models [16]. Hence, there is much incentive for the “in-house laboratory” development of physiologically relevant 3D skin models for preclinical testing. Given the prerequisite to explore the use of novel vitamin D derivatives in skin care [5], and given the efficacy of the VD3/VD2+AgNP combination in wound healing on HaCaT cells [11,14], the objective of the present study was to validate the efficacy of VD2+AgNP in wound healing using a 3D reconstructed human skin (RHS) equivalent documented to be effective in disease research as well as pharmacological and cosmetic testing [17]. The model was composed of healthy primary skin cells from foreskins, developed by Truzzi et al. [18]. Of interest, in recent reviews on the innovative use of natural ingredients in wound-healing as well as in sunscreens, no mention of vitamin D was made [2,19]. Hence, the aim of the present study was extended to evaluate the potential multi-purpose efficacy of VD2+AgNPs in UVB protection and skin irritation. This was considered relevant since there is a demand for innovative alternatives in the photoprotective skincare industry.

## 2. Materials and Methods

### 2.1. Materials

The VD2 agaric extract and the AgNPs (more than 80% with an average size of approximately 100 nm) were obtained from ALIDANS SRL (San Giuliano Terme, Pisa, Italy) and the NanoBMat Company (Düsseldorf, Germany), respectively. Primary cells, composed of keratinocytes and fibroblasts, were isolated from neonatal foreskins. The foreskin material was obtained after obtaining signed informed consent. Dulbecco’s Modified Eagle Medium (DMEM), Fetal Bovine Serum (FBS), L-Glutamine, Penicillin–Streptomycin, rat tail collagen type I and Hanks’ Balanced Salt Solution (HBSS) were purchased from GIBCO (Waltham, MA, USA); 3-(4,5-dimetiltiazol-2-il)-2,5-difeniltetrazolio (MTT) was obtained from Life Technologies (Carlsbad, CA, USA). The hematoxylin and eosin (H&E) stain was purchased from Bio-Optica^®^ (Milan, Italy). Reagents for the immunohistochemical analysis included involucrin, Ki67 and the ImmPRESS HRP Horse Anti-Rb IgG Polymer Detection Kit, which were purchased from ABclonal Technology (Düsseldorf, Germany), Novus Biologicals (Centennial, MO, USA) and Vector Laboratories Inc. (Newark, CA, USA), respectively. The Human IL-1α ELISA FineTest kit was obtained from Wuhan Fine Biotech (Limerick, PA, USA). All other chemicals and solvents were of analytical grade.

### 2.2. Preparation of Vitamin D2 and Silver Nanoparticle Cream Extract

The extraction protocol for obtaining the vitamin D2 is described in the instructions provided by ALIDANS S.R.L (San Giuliano Terme, Italy). The extraction process was based on adding the powdered mushroom extract to sunflower oil and tocopherol. The maceration process was then allowed to proceed for an unspecified time, after which, the VD2 was titrated. At the end of the process, the agaric extract in sunflower oil and tocopherol was standardized to a concentration of VD2 of around 20 mg/kg (800 IU/g). This extract served as one of the primary ingredients to which the other ingredients were then added. The final cream preparation was an emulsion containing the following: water, *Helianthus annuus* seed oil, *Agaricus bisporus* extract, sorbitan isostearate, polyglyceryl-3 polyricinoleate, hydrogenated castor oil, magnesium stearate, magnesium sulfate, tocopherol, colloidal silver, caprylyl glycol, 1,2-hexanediol and hydroxyacetophenone. Sorbitan isostearate and polyglyceryl-3 polyricinoleate served as the emulsifiers. The ingredients were mixed together according to a standardized protocol by the Kalis dermocosmetic laboratory in Treviso, Italy. However, all information pertaining to the stages in the production process is confidential. The final ingredients of the cream were 6.3 µg/mL VD2 + 5 µg/mL di AgNPs. The concentrations chosen were based on previous work [11,14] and were not shown to be toxic to HaCaT cell viability.

### 2.3. Construction of the Reconstructed Human Skin Model

Normal human keratinocytes and fibroblasts were isolated from 13 separate neonatal foreskin samples and cultured in DMEM and Ham’s F12 media with serum, as described by Pincelli et al. [20]. Total keratinocytes were cultured in DMEM for use in the construction of the RHS models, as described in Truzzi et al. [18]. Briefly, cell-free collagen solution (1.35 mg/mL rat tail collagen type I in DMEM with 10% FBS and 1% Pen/Strep) at a volume of 0.5 mL was added to 12-well tissue culture inserts (Transwell, Costar, Cambridge, MA, USA). This precoated layer was overlaid with 1 mL of primary fibroblasts (15 × 10^4^ cells) mixed with type I collagen solution. After 4 days of incubation at 37 °C, dermal reconstructs were rinsed with keratinocyte medium [20] for 1 h at 37 °C. The total keratinocytes (25 × 10^4^ cells) were seeded on dermal reconstructs, incubated for 1 h at 37 °C and then submerged in keratinocyte medium [20] for 4 days. Finally, skin reconstructs were exposed to the air; the keratinocyte medium was changed every two days.

After 12 days, the RHS models were exposed to wound healing, UVB irradiation and irritation treatments, respectively, in either the absence or in the presence of topical VD2+AgNP cream. For each treatment, 3 separate replicates (each representing a separately constructed model from an individual foreskin sample) were used. All treated models were incubated with 500 µL topical cream containing VD2+AgNPs at the correct concentrations, reported previously [11,14]. Untreated models were not exposed to VD2+AgNPs, but were instead incubated with 500 µL medium, the volume required to cover the surface of the models.

### 2.4. Wound Healing Measurements on Reconstructed Human Skin Models

Mechanical wounding was implemented based on the method of Rodriguez et al. [21]. Then, the medium was aspirated from each well and a vertical cut (scratch line) was made with the tip of a steel pin to produce a slash that penetrated both the upper keratinocyte layer and the underlying dermal layer. Experimental models were incubated with 500 µL topical cream, whereas the untreated models were not exposed to the treatment. Models were incubated for 24 h.

The wound healing assay was similarly performed using a biopsy punch with a diameter of 2 mm to create a larger circular injury. Experimental models were similarly incubated with 500 µL topical cream containing VD2+AgNPs. The models were incubated for 48 h and 72 h in the presence and absence of treatments. The plates were visualized under an optical microscope (Eclipse Ts2, Nikon, Boston Industries, Walpole, MA, USA) at a magnification of 20× and 40× and photographed. Models were also paraffin-embedded and stained with H&E and examined under light microscopy (Section 2.6). Wound healing was assessed microscopically from the progress of cell proliferation and migration into the wound site to reduce the wound gap over time, and compared to the respective untreated CTRLs. For each sample analyzed, measurements were made from 10 different areas. The final results were expressed as the mean of three different experiments.

### 2.5. Skin Irritation and UVB Irradiation Treatments on Reconstructed Human Skin Models

For the skin irritation studies, 5% sodium dodecyl sulphate (SDS) was selected as a skin irritant based on the Organization for Economic Co-operation and Development Test Guidelines 439 (OECD TG 439) [22]. Following the pretreatment of 500 µL topical cream containing VD2+AgNPs to RHS models for a 30 min incubation period, 5% SDS was added to both the experimental and CTRL models, which were then post-incubated for 4 h at 37 °C in a humidified atmosphere containing 5% CO_2_.

The photoprotective effect of the topical cream (containing VD2+AgNPs) in response to UVB radiation was investigated on the RHS models using two approaches: In the first, 500 µL topical cream was administered to the models and incubated for 1 h prior to UVB exposure. In the second approach, 500 µL topical cream was administered immediately after UVB exposure. This was then followed by a 4 h post-irradiation period. The comparative CTRL models (minus topical cream) were subjected to the same procedure for each respective approach. UVB irradiation was 50 mJ/cm^2^ using a Philips PL-S 9W/01/2P light source (Signify commercial UK Limited, Guildford Surrey GU2, UK).

### 2.6. Morphological Assessment and Immunohistochemical Detection of Involucrin and Ki67

The RHS models were paraffin-embedded and stained with H&E according to Truzzi et al. [22]. Sections (4 μm thick) were visualized using light microscopy (MEIJI Techno Co., Ltd., San Jose, CA, USA) at a magnification of 40× and photographed. Morphological evaluation included measurements of epidermal thickness and percentage detachment of the epidermis from the underlying dermis.

For the immunohistochemical detection of involucrin and Ki67, the ImmPRESS HRP Horse Anti-Rb IgG Polymer Detection Kit was used according to the manufacturer’s instructions. Briefly, the 4 μm sections were incubated with primary antibodies for 1 h at 37 °C or overnight at 4 °C for involucrin and Ki67, respectively. Subsequently, ImmPRESS horse anti-rabbit and anti-mouse secondary antibody solution was added. This was followed by adding 3,3′-Diaminobenzidine (DAB) staining and hematoxylin re-staining of the cell nucleus. Quantification of involucrin and Ki67 was performed by calculating the percentage of positive pixels on micrographs. To perform the analysis of the pixels, digital images were processed to 300 pixels/inch and converted to 8 bits. The binary images were then further processed by the “color deconvolution” plugin to analyze the staining of the marker of interest. The selected picture was saved as a tiff for a “clean-up” procedure to eliminate artefacts with Adobe Photoshop CC (version 20.0.4). Thereafter, all fields of interest were measured with the application “Analyze particle” of ImageJ2, and the data were reported as the number of pixels. Each experiment was performed in triplicate, with three internal replicate fields analyzed for each replicate.

### 2.7. Viability Measurements

The viability of the keratinocytes and fibroblasts in the RHS models was measured using MTT, according to Kandárová et al. [23]. The percentage of cell proliferation was calculated using the following formula: absorbance value of treated sample/absorbance value of control × 100 = % of cell viability.

### 2.8. Determination of Interleukin 1Alpha

After the skin irritation experiments, the medium was removed from the models for the measurement of the inflammatory cytokine, Interleukin 1alpha (IL-1α). IL-1α was measured using the Human IL-1α ELISA FineTest kit according to the manufacturer’s instructions.

### 2.9. Statistical Analyses

The data was expressed as the mean values of the three different experiments. Statistical analysis was conducted using GraphPad Prism Version 10.4.1 (2024). One-way variance (ANOVA) was used to determine any significant differences between the respective treatments compared to the CTRL. Using Dunnett’s multiple comparisons test, significant differences were represented as follows: not significant (ns), * *p* < 0.05, ** *p* < 0.01, *** *p* < 0.001, and **** *p* < 0.0001. In the graphs, mean values expressed with stars are statistically different.

## 3. Results and Discussion

**Figure 1:**
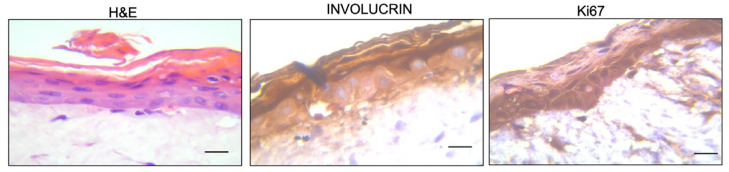
Hematoxylin and Eosin (H&E) staining and immunohistochemical staining of involucrin and Ki67 at 40× magnification of the two-layered (epidermal and dermal) reconstructed human skin model. The scale bar is 50 µm.

The RHS equivalent used in the present study represented an improvement over the more commonly used RHE model (containing only keratinocytes on an inert matrix). The two-layered full-thickness skin equivalent, composed of both epidermal and dermal skin layers, provided a more complex model of human skin. A more complex model is considered more effective in wound healing studies and advanced dermatological research [16], as well as in evaluating topical ingredients for preclinical studies [24]. The efficacy of our model in advanced dermatological research was validated previously [18,25]. Given that the present model was used the first time to evaluate the effects of a topical cream, it was necessary to demonstrate the efficacy of the model as an experimental platform. This was demonstrated with H&E, involucrin and Ki67 staining (Figure 1).

Basic morphology with H&E staining showed the stratified epidermis in the RHS equivalent after the 12-day developmental period (Figure 1). The stratum basal (SB) and the stratum spinosum (SS) were shown with clearly visible blue-stained nuclei. Also evident were the pink-stained anucleate cornified layers (or corneocytes) forming the stratum corneum (SC). The detachment of the outermost corneocyte layers was evident, illustrating the physiological desquamation process (Figure 1). Beneath the dermal–epidermal junction (DEJ), the dermis with human dermal fibroblasts and fibroblast-producing collagen fibers in the extracellular matrix (ECM) are shown (Figure 1). The structure of the present RHS equivalent was shown to be suitable for topical cream testing by comparisons to previous H&E-stained RHS equivalents [26,27].

The efficacy of the present model was also verified from the expression of markers of epidermal proliferation and differentiation. The terminal differentiation marker, involucrin, is synthesized in the SS and cross-linked in the stratum granulosum (SG) by the transglutaminase enzyme to produce the keratinocyte cornified envelope. Immunohistochemical staining of involucrin was strongly positive (in brown) in the SS, SG and, more specifically, the SC layers (dark brown) (Figure 1). In contrast, the immunohistochemical staining of Ki67-stained nuclei (dark brown) was restricted to the actively proliferating SB layer of the epidermis (Figure 1). The presence of the Ki67-stained SB layer and the involucrin-stained SS, SG and SC layers, respectively, have been similarly demonstrated in RHS models that are considered comparable to human skin [27,28].

The topical cream formulation containing 133 nm VD2 and 5 ppm AgNPs, in accordance with the concentrations stabilized by Cataldi et al. [11] and Ruggiero et al. [14], was then investigated on models subjected to wound injury, skin irritation treatment with 5% SDS and 50 mJ/cm^2^ UVB irradiance.

### 3.1. Wound Healing Efficacy of the of Vitamin D2 and Silver Nanoparticle Cream Extract

**Figure 2:**
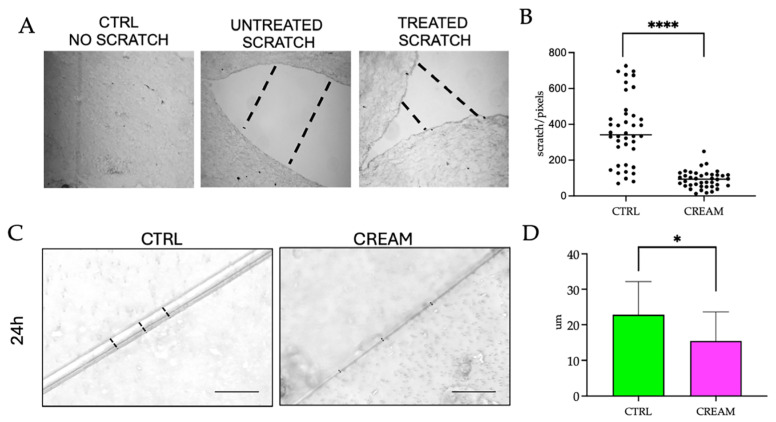
(**A**) Optical microscopic images of the paraffin-embedded sections at 40× magnification of the control model (CTRL), an untreated wound-scratched border and topical vitamin D2 + silver nanoparticle (VD2+AgNP) cream on a wound-scratched border after a 24 h incubation. (**B**) Statistical analyses of the untreated and cream-treated scratch border after 24 h. The black dots indicate the distance positioning of the individual replicates within the wound border. (**C**) Optical microscopic images comparing the surface diameter of the untreated and cream-treated scratch after 24 h at 20× magnification. The hatched lines in the images indicate the distance of the gap created by the incision close to the wound border (**A**) and along the length of the scratch (**C**). (**D**) Statistical analyses of the mean and standard deviation of the untreated and cream-treated scratch length diameter after 24 h. Significant differences were reported as follows: * *p* < 0.05, **** *p* < 0.0001.

Since the VD3/VD2+AgNP-containing creams have been previously shown to induce the reparative activity of HaCaT cells in 2D [11,14], the first objective was to validate the wound healing efficacy of VD2+AgNPs on the 3D model. To this end, re-epithelialization, the defining parameter of successful wound closure [28], was measured from epidermal keratinocyte cell-migration and proliferation into the wound gap. Wounding was first implemented by a mechanical scratch. Administration of the VD2+AgNP cream was shown to significantly decrease the distance within the wound gap of the scratch wound border compared to that of the untreated wound after 24 h (Figure 2A). Although keratinocyte migration was shown previously to be initiated within 24 h (without the presence of growth factors) in both untreated superficial (epidermis) and full-thickness (epidermis and dermis) injuries [29], the components contained in the cream significantly stimulated a more rapid keratinocyte migration within the 24 h period (Figure 2B). In addition to the wound border, re-epithelialization was investigated along the length of the scratch (Figure 2C). Similarly, the distance between the two margins was significantly lower after application with VD2+AgNPs compared to the untreated wound (Figure 2D), although repair was not as rapid as at the wound edge region (Figure 2B).

**Figure 3:**
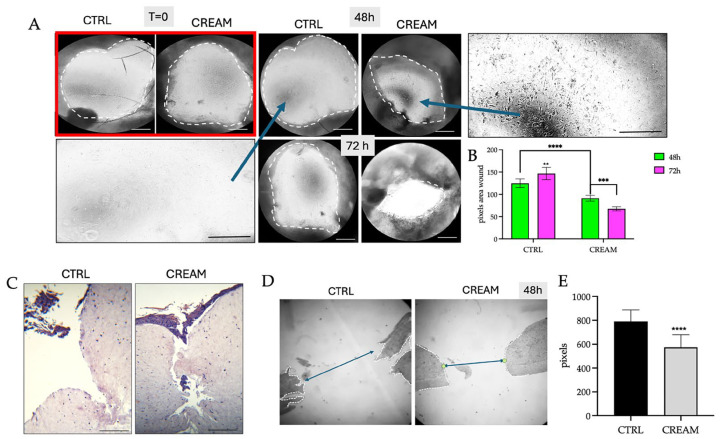
(**A**) Optical microscopic images (20× magnification) of reconstructed human skin models comparing the untreated (CTRL) and topical vitamin D2 + silver nanoparticle (VD2+AgNP) cream treatments at 0 (T = 0), 24 and 48 h after mechanical punch injury. The white hatched lines indicate the wound margins. Inlet: comparison of fibroblast migration in the dermal extracellular matrix within the wound area between the untreated CTRL and cream-treated models after 48 h. (**B**) Statistical analysis of the wound area in the CTRL and topical cream-treated models at 48 and 72 h after wounding. (**C**) Paraffin-embedded histological cross-sections of the full-thickness wound penetrating both the epidermis and dermis and (**D**) keratinocyte migration in the CTRL and topical cream-treated models, respectively, at 48 h post-wounding. The scalebar is 200 µm. (**E**) Statistical analysis of the histological images depicting wound area in untreated and topical cream-treated wounds after 48 h. (**B**,**E**) Results were reported as the mean ± SD and significant differences were reported as follows: ** *p* < 0.01, *** *p* < 0.001, **** *p* < 0.0001.

Since blade or steel scratchers create narrower scratches and result in a faster scratch closure rate, which is challenging to assess [30], the wound healing capacity was also investigated following a biopsy punch to penetrate both the epidermis and underlying dermis, evident in Figure 3. Moreover, the analysis was extended to a longer 48–72 h period, in which not only inflammatory-induced keratinocyte migration but also the proliferation phase of wound healing is reported [31]. The circumference of the punch wound was shown to be significantly reduced in the topical cream-treated models after 48 h compared to the untreated CTRL (Figure 3A,B). After 72 h, the wound area of the VD2+AgNP-treated model was significantly reduced compared to 48 h (Figure 3B). Paraffin-embedded tissues, similarly, reflected a significantly decreased distance between wound margins after 48 h (Figure 2D,E).

At time 0, there were no fibroblasts visible in the CTRL and the topical cream-treated model (Figure 3A). After 48 h, migrating fibroblasts in the underlying exposed dermis were evident following exposure to the topical cream. This was not evident in the CTRL (Figure 3A). The lack of fibroblasts in the dermis of untreated models following a full-thickness injury has been documented previously [29]. The cream treatment was shown for the first-time to induce a stimulatory effect on fibroblast migration in the dermis. Given that the combination of both components was used, it was not possible to establish the individual contributions of VD2 and AgNPs to dermal fibroblast migration, as was demonstrated previously for keratinocyte migration [11,12].

AgNPs were previously shown to stimulate dermal fibroblast migration in either 2D or in vitro full-thickness rat skin models [32,33,34]. Instead, to the best of our knowledge, there are no reports documenting the migration of fibroblasts in response to the topical application of vitamin D after wound healing [5]. Interestingly, the keratinocyte migration rate was previously shown to increase significantly when in the presence of fibroblasts. The keratinocyte cells were able to reach the center of the wound at twice the rate compared to that in the absence of fibroblast co-cultures [35].

Keratinocyte re-epithelialization is strictly dependent on the interaction between keratinocytes and dermal fibroblasts. The proliferation of keratinocytes is dependent on the fibroblast synthesis of paracrine factors and on the restoration of the DEJ, which becomes torn in full-thickness wounds (Figure 3C) [29]. Fibroblasts are also responsible for the synthesis of collagen into the wound and are able to differentiate into myofibroblasts, resulting in the contraction of the wound bed to facilitate keratinocyte re-epithelialization [29,36,37]. Noteworthily, during wound healing, AgNPs have been shown to promote wound contraction by stimulating the differentiation of fibroblasts into myofibroblasts [32,33]. This aspect warrants further investigation.

### 3.2. Protective Efficacy of the of Vitamin D2 and Silver Nanoparticle Cream Extract in Skin Irritation

The detergent SDS is a well-known inducer of irritant contact dermatitis. As such, the OECD TGs [22] stipulate that a concurrent negative CTRL and positive CTRL of 5% aqueous SDS be used in comparative assessments with the novel substances under investigation (cream + 5% SDS).

**Figure 4:**
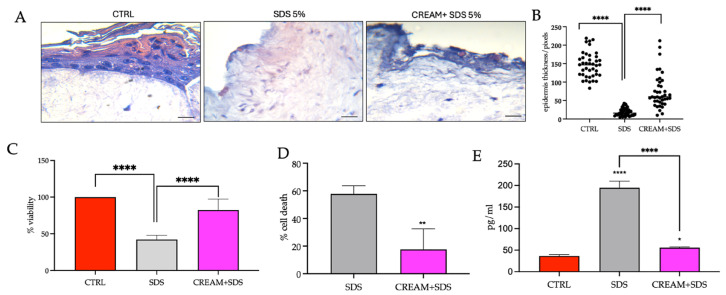
(**A**) Hematoxylin and eosin (H&E) staining at 40× magnification of the two-layered (epidermal and dermal) reconstructed human skin model control (CTRL), the 5% sodium dodecyl sulfate (SDS) and the cream + SDS models (VD2+AgNP cream incubated for 30 min prior to SDS exposure), respectively, following a 10 min exposure to 5% SDS and a 4 h post-incubation period. The scale bar is 50 µm. (**B**) Epidermal thickness measurements from microscopic images with the black dots indicating the thickness of individual replicates along the length for the epidermis. (**C**) The percentage viability (MTT assay), (**D**) cell death and (**E**) Interleukin 1alpha levels in the CTRL, 5% SDS and cream-SDS models following the incubated periods indicated above. Results are expressed by the mean and standard deviation, with significant differences reported as follows: * *p* < 0.05, ** *p* < 0.01, **** *p* < 0.0001.

Given that irritation is the production of reversible skin damage, models were exposed to 5% SDS for 10 min and examined post-incubation after 4 h, with the objective of investigating rapid protective efficacy over a short time period. The exposure time for the positive control varies depending on the reconstructed human epidermis model used. In the present study, it was observed that with our model, 10 min was sufficient to induce damage without destroying the model. As regards selecting a 4 h post-incubation period, we observed that with in vitro monolayer studies it was possible to visualize repair or damage effects on skin samples after 4 h. In comparison to the CTRL with well-defined SB, SS, SG and SC layers, the addition of 5% SDS was shown to impact the structure of the epidermis, showing a significantly reduced or absent SC (Figure 4A). This was verified by the significantly reduced overall epidermal thickness (Figure 4B). The present results corroborated previous research, showing that 0.1% SDS over a period of 20 h resulted in the complete disappearance of the outermost cornified layer, and the breakage of the cornedesmosomes that hold the innermost layer of corneocytes of the SC [38]. A significant decrease in SC thickness was also recorded [39]. Protection afforded by the VD2+AgNP cream treatment, administered 30 min prior to the irritant, was evident. Epidermal thickness was ameliorated in comparison to the SDS treatment (Figure 4A,B). The OECD Guideline No. 439 is designed to evaluate the irritancy potential of chemicals using reconstructed human epidermis models. However, it does not specify protocols for testing the effectiveness of barrier creams against skin irritation. Therefore, to evaluate a cream with a protective function, the skin model can be exposed to the irritant in the presence or absence of the barrier cream, but the required exposure time to the cream is not specified. We chose 30 min as a reasonable time required to protect the skin of a child before exposure to the irritant.

The cell viability of the CTRL was set to 100%. The administration of the 5% SDS for 10 min was shown to reduce the viability of the cells in the combined epidermal and dermal cell layers to approximately 40% over the 4 h post-incubation period (Figure 4C,D). This value was significantly higher than the 2–10% viability recorded previously following exposure to 5% SDS [27,28,39,40,41]. However, in the latter case, SDS exposure ranged between 45–60 min, followed by a 42 h post-incubation period, which resulted in more damage [41]. Moreover, in some reports, viability was only assessed in commercially available epidermal models. These models did not take into account the dermal fibroblasts [39,40]. The viability of the dermal fibroblasts was suggested to be under-estimated in a laboratory-generated full-skin model [27]. Administration of VD2+AgNPs for 30 min, prior to exposure to the irritant, afforded a 40% protection against SDS. The viability of the cream-treated models declined by only 20% in comparison to the CTRL (Figure 4C,D).

IL-1α, a widely used early marker for skin irritation, is constitutively expressed in keratinocytes and is only released from cells with disrupted membranes. This cytokine serves as the initial “alarm signal” to surrounding cells, resulting in the production of additional cytokines in the inflammatory phase of wound healing [41,42]. After SDS detergent application, IL-1α was significantly increased in the broken keratinocytes and was 5.5-times higher than the CTRL (Figure 4E). Previous results showed a 1.5–4.0 increase in IL-1α in commercially available 3D models, containing only keratinocytes, after 42 h exposure to 5% SDS compared to the CTRL [40,41].

The potential implication of using VD2+AgNP, not only in wound healing but also in irritative pathologies, has been raised previously [14]. The present study showed the combined preventative efficacy of VD2+AgNP against skin irritation in a 3D skin equivalent for the first time. The rapid development of nanotechnology and the use of NPs has led to concern over the deleterious exposure to humans and the environment. As such, previous research has focused on nanoparticles as potential skin irritants. However, AgNPs alone have been increasingly recognized as non-toxic in skin irritation testing [39,43] and can, therefore, be considered suitable in facilitating the targeted topical delivery of VD2 against skin irritation.

### 3.3. Protective and Curative Efficacy of the Vitamin D2 and Silver Nanoparticle Cream Extract in Ultraviolet B Irradiation

**Figure 5:**
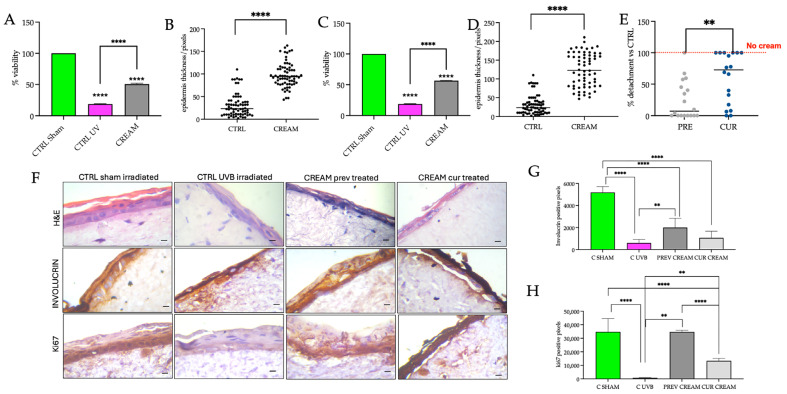
(**A**) Viability (MTT assay) and (**B**) epidermal thickness measurements made from Hematoxylin and eosin (H&E)-stained images comparing the control (CTRL) sham-irradiated models, the CTRL UVB-irradiated models (exposed to 50 mJ/cm^2^) and the cream models (cream applied 1 h prior to UVB exposure) after a post-irradiation period of 4 h. (**C**) Viability (MTT assay) and (**D**) epidermal thickness measurements made from H&E-stained images comparing the CTRL sham, UVB-irradiated and cream (cream applied immediately after irradiation) models after a post-irradiation period of 4 h. (**E**) Comparison of the epidermal thickness between the Prev (preventative) cream applied 1 h prior to UVB exposure) and Cur (curative, cream applied immediately after irradiation). The black dots represent individual measurements. (**F**) H&E, involucrin and Ki67 staining at 40× magnification of the reconstructed human skin (RHS) models. Comparisons were between the CTRL sham-irradiated, CTRL UVB-irradiated, the cream Prev and the cream Cur models, respectively, after a post-irradiation period of 4 h. The scalebar is 200 µm. Statistical analysis of (**G**) involucrin and (**H**) Ki67 expression from the histological images. All results were reported as the mean ± SD and significant differences are reported as follows: ** *p* < 0.01, **** *p* < 0.0001.

No mention of Vitamin D was made in connection with the use of natural ingredients in sunscreens [19], for which there is a demand in the photoprotective skincare industry [19]. Aside from the promising potential of VD2+AgNP in wound healing and skin irritation protection, we decided to examine both the damage prevention (protective efficacy) and the curative efficacy of the topical cream in RHS equivalents exposed to narrow-band UVB (290–320 nm). Narrow-band UVB is almost completely absorbed by the epidermis, with comparatively little reaching the dermis [44]. The biologically efficient dose (BED) of UVB capable of inducing early UVB damage in an RHS equivalent has been established at 50 mJ/cm^2^ [45]. Therefore, 50 mJ/cm^2^ was used in the present study. The protective efficacy of the cream was investigated by administering the VD2+AgNP to the models for 1 h prior to UVB exposure. It is recommended that creams be administered at least 15–30 min prior to sun exposure in small children. Hence, the selection of 1 h prior to exposure was considered sufficient to protect the delicate skin of young children. After a 4 h post-irradiation period, cell viability (Figure 5A), epidermal thickness (Figure 5B) and percentage detachment of the epidermis (Figure 5E), as well as involucrin (Figure 5F,G) and Ki67 (Figure 5F,H) expression, were examined. The curative efficacy was examined by administering the cream immediately following UVB exposure. Similarly, after the 4 h post-irradiation period, vitality (Figure 5C), epidermal thickness (Figure 5D), percentage detachment of the epidermis (Figure 5E) and involucrin (Figure 5F,G) plus Ki67 (Figure 5F,H) expression, were determined.

Viability in UVB-treated models declined to 15% compared to the CTRL sham-irradiated models (set to 100% viability; Figure 5A,C). The present results corroborated the cell viability percentages recorded previously in the Episkin model (composed of a fibroblast-populated dermal equivalent and a fully differentiated epidermis) after 40 mJ/cm^2^ UVB exposure [46]. Administration of the VD2+AgNP cream, either prior to irradiation (Figure 5A) or immediately after (Figure 5C), restored viability to approximately 50% of the CTRL sham-irradiated models. UVB damage, resulting in the detachment of the epidermis in the untreated UVB-irradiated models, was set to 100% (Figure 5E) and used to determine the comparative efficacy of the respective cream-treated models. The higher protective efficacy of the cream, compared to a curative efficacy, was demonstrated from a significantly lower comparable percentage detachment of the epidermis (Figure 5E). A large variation in epidermal detachment was evident for the curative experimental treatment. The reduced epidermal thickness of the untreated UVB-treated models reflected epidermal breakage. Both cream treatments increased epidermal thickness, with the curative treatment showing a higher response variation (Figure 5B,D).

Damage resulting in epidermal breakage is attributable to many factors. Aside from being a marker of cornified envelope production, involucrin is considered to be a pivotal factor in preserving the functional integrity of the skin barrier [47]. Untreated UVB-irradiated models showed a significant decrease in involucrin expression (Figure 5F,G), corroborating previous research showing significant decreases in HaCaT keratinocytes or reconstructed models following UVB exposure in the ranges between 2 and 50 mJ/cm^2^ [48,49,50]. The effect of UVB irradiation on Tight Junction (TJ) integrity, not measured in the present study, cannot be excluded. Loss of TJ integrity and protein content was reported previously in a 40 mJ/cm^2^ UVB-irradiated epidermis [51] and in 50 mJ/cm^2^ UVB-irradiated HaCaT cells [52], respectively. Based on the expression of the proliferation marker Ki67, the proliferative capacity of the SB was significantly reduced in the untreated UVB-irradiated models (Figure 5F,H). Previous research using unpigmented RHS models (with both epidermal and dermal components) also showed a significant reduction in Ki67 expression after UVB irradiation [53,54]. Ki67 was then shown to be upregulated once again after 3–4 days in the regenerative phase [53].

Administration of the topical cream in a damage-preventative capacity increased involucrin and Ki67 to a significantly greater extent than that evident when cream was administered directly after UVB irradiation (Figure 5F–H). Of additional interest was the presence of numerous fibroblasts close to the DEJ in the H&E images of VD2 + AgNP cream (damage prevention capacity) models (Figure 5F). This was also shown in the wound healing response (Figure 3A). In contrast, although fibroblasts were present in the curative cream models, these were not shown to have migrated close to the DEJ. Overall, the present study indicated for the first time that VD2+AgNP in combination was more effective at minimizing UVB radiation damage in a protective capacity. Individually, both Vitamin D and AgNPs were previously shown to protect skin from UVB irradiation [55,56,57].

## 4. Conclusions

Using preclinical RHS equivalents, the present results validated the efficacy of the VD2+AgNP cream on wound healing, shown previously for human keratinocyte cells [11,14]. The VD2+AgNP cream was also shown to stimulate fibroblast migration in the wounded dermis, important in promoting re-epithelialization of the DEJ and keratinocytes. In addition to wound healing, the VD2+AgNP cream showed effectiveness in ameliorating irritation induced by 5% SDS. Compared to the 5% SDS-treated model, the cream + SDS showed a significant increase in viability, affording protection up to 40%. Protection was associated with increased epithelial thickness and decreased pro-inflammatory IL-1α expression. UVB damage resulted in a significant reduction in viability, increased epidermal detachment and consequent reduced epidermal thickness. UVB damage also resulted in decreased involucrin (in the SC, SG and SS layers) and Ki67 (in the SB layer) expression. Administration of the topical cream prior to UVB irradiation was shown to be more effective than the administration of the cream following UVB irradiation in minimizing UVB-induced damage. The efficacy of the preventative cream was demonstrated from the lower detachment percentage and significantly higher involucrin and Ki67 expression compared to the curative treatment. The combination of VD2+AgNP was shown for the first time to be effective as a protectant against skin irritation and UVB irradiation. The underlying molecular mechanisms were not investigated and warrant research attention in order to further promote the use of VD2+AgNP in the skin repair and sun protection industries.

## Data Availability

Dataset available on request from the authors.

## Abbreviations

The following abbreviations are used in this manuscript:

2D	Two-Dimensional
3D	Three-Dimensional
AgNPs	Silver Nanoparticles
CTRL	Control
DEJ	Dermal-Epidermal Junction
DMEM	Dulbecco’s Modified Eagle Medium
ECM	Extracellular Matrix
FBS	Fetal Bovine Serum
HBSS	Hanks’ Balanced Salt Solution
H&E	Hematoxylin and Eosin
IL-1α	Interleukin 1alpha
RHS	Reconstructed Human Skin
SB	Stratum Basal
SC	Stratum Corneum
SG	Stratum Granulosum
SS	Stratum Spinosum
SDS	Sodium Dodecyl Sulfate
USD	United States dollar
UVB	Ultraviolet B light
VD2	Vitamin D2
VD3	Vitamin D3
